# An Insight into In Vitro Antioxidant, Antimicrobial, Cytotoxic, and Apoptosis Induction Potential of Mangiferin, a Bioactive Compound Derived from *Mangifera indica*

**DOI:** 10.3390/plants12071539

**Published:** 2023-04-03

**Authors:** Ramy S. Yehia, Sarah A. Altwaim

**Affiliations:** 1 Department of Biological Sciences, College of Science, King Faisal University, Al-Ahsa 31982, Saudi Arabia; 2 Department of Botany and Microbiology, Faculty of Science, Cairo University, Giza 12613, Egypt; 3 Department of Medical Microbiology and Parasitology, Faculty of Medicine, King Abdulaziz University, Jeddah 21589, Saudi Arabia; 4 Special Infectious Agents Unit, King Fahd Medical Research Center, King Abdulaziz University, Jeddah 22252, Saudi Arabia

**Keywords:** bioactivities, mangiferin, characterization, *Mangifera indica* L.

## Abstract

Due to their low cost, toxicity, and health risks, medicinal plants have come to be seen as useful products and sources of biologically active compounds. *Mangifera indica* L., a medicinal plant with a long history, has a high bioactive metabolites content. Mangiferin (C_19_H_18_O_11_) is primary isolated from *M. indica*’s leaves, which has many pharmacological benefits. In this investigation, ultrasonic-assisted extraction with ethanol as the extraction solvent was applied to obtain mangiferin from a local type of *M. indica* leaves. HPLC was performed after a dichloromethane-ethyl acetate liquid–liquid fractionation method. Further, UV–vis, FTIR, and NMR spectroscopy were utilized to elucidate the structure. Interestingly, purified mangiferin displayed promising antimicrobial efficacy against a diverse variety of fungal and bacterial pathogens with MICs of 1.95–62.5 and 1.95–31.25 µg/mL, respectively. Time–kill patterns also showed that mangiferin had both bactericidal and fungicidal action. Furthermore, it exhibited strong radical dosage-dependent scavenging activity (IC_50_ = 17.6 μg/mL) compared to vitamin C (Vc, IC_50_ = 11.9 μg/mL), suggesting it could be developed into a viable antioxidant agent. To our delight, the IC_50_ values of mangiferin for the MCF-7 and HeLa cell lines were 41.2 and 44.7 μg/mL, respectively, from MTT cell viability testing, and it was less harmful when tested against the noncancerous cell line. Notably, it significantly induced cell apoptosis in MCF-7 cells by 62.2–83.4% using annexin V-FITC/PI labeling. Hence, our findings suggest that mangiferin can be used in the medical industry to create therapeutic interventions and medication delivery systems for society.

## 1. Introduction

Numerous communities employ herbal medicines derived from medicinal plants to treat and avoid a wide range of illnesses [1]. Plant barks, leaves, flowers, and other organs have been utilized in medicine. These similar chemicals found in plants have just lately been used to generate synthetic pharmaceuticals [2]. Medicinal plants are a significant source of biologically active natural chemicals and are used as an alternate and/or supplementary treatment method due to their extensive pharmacological, therapeutic, and other biological properties [3].

Given the recent increase in microbial infections in humans, scientists have turned their attention to medicinal plants as low-cost and efficient forms of treatment [4]. Due to the development of microbial resistance to numerous antibiotics, the utilization of extracts and bioactive chemicals produced from medicinal plants as resistance to bacteria has expanded [5]. Plant-based medicines are garnering popularity because of their minimal toxic effects and negligible health consequences [6].

Natural substances exhibit remarkable antioxidant action. Although increasing oxidative stress is believed to play a significant role in the proliferation and evolution of numerous high-risk diseases in humans, the antioxidant capacity of bioactive substances is of immense importance [7]. Hence, the natural phytochemicals found in medicinal plants may serve as a source of treatments with varying degrees of antimicrobial activity, sometimes even at low dosages [8].

Drug-resistant diseases will disproportionately affect low-income communities. In an attempt to fill this information gap, *Mangifera indica* L., a traditionally used medicinal plant from the Anacardiaceae family, was explored to uncover active biological compounds that might enhance potentially antioxidant and antibacterial capabilities [9]. Several *Mangifera* species have been discovered to offer therapeutic benefits, including antidiabetic [10], antiviral [11], antibacterial [12], anti-Alzheimer agent [13], antioxidant [14], and anticancer [15]. Furthermore, *M. indica* has a wide range of bioactive compounds, including vitamins A and C, protein, carotenoids, benzoic acid, gallic acid, carbohydrates, fiber, minerals, and phenolic compounds (such as iriflophenones, quercetin, catechin, and gallotannins) [16,17]. Several pharmacological actions are hypothesized to originate from these bioactive molecules.

Mangiferin (C_19_H_18_O_11_), a natural glucoxanthone, is one of the major bioactive compounds present in different parts of *M. indica*, including the leaves, barks, and peels, as well as many other plants [18]. Mangiferin has been shown in numerous studies to have a wide spectrum of biological actions, making it a viable agent for the food and pharmaceutical sectors. It offers several health-promoting properties, such as anti-inflammatory, antiviral, immunoregulatory, and anticancer capabilities [19].

To date, fewer investigations have clarified the pharmacological and phytochemical properties of *M. indica* in Eastern Province, Saudi Arabia. In this context, advanced procedures were used to extract and identify mangiferin from a local type of *M. indica* in order to initiate the current inquiry. Several spectroscopic analyses, including UV–vis, FTIR, NMR, and HPLC, were employed for its characterization. Antimicrobial, antioxidant, cytotoxic, and protective properties were evaluated in vitro in order to ascertain their therapeutic relevance.

## 2. Results and Discussion

Plants are a major source of natural chemical scaffolds that are used as models to create new bioactive compound. By employing ultrasonic extraction with ethanol as an extraction solvent, mangiferin from local mango leaves was recovered in the current investigation. Mangiferin was previously extracted using traditional techniques such as Sohlex, heat reflux, and maceration extraction [20]. The chemical produced was, however, quite sensitive to the operating conditions, and these procedures required a large amount of time and solvents [21]. Numerous research studies have examined the efficacy of mangiferin extraction from diverse sources in the context of the recent advancement of novel extraction techniques such as subcritical fluid, ultrasonic extraction, and microwave-assisted extraction [22,23,24]. For instance, mangiferin was extracted using ultrasonic extraction alone and in combination with three-phase partitioning, with corresponding extraction efficiencies of 1.27, 41, and 58.46 mg/g [25,26]. Here, ultrasonic waves at frequencies higher than 20 kHz were utilized to disturb cells, which improved the solvent’s ability to penetrate. Hence, ethanol was suggested as a nontoxic solvent since it yielded the highest content of mangiferin. In consequence, an ultrasonic extraction method provided a rapid extraction technology of mangiferin, demonstrating its economic viability. In the future, it can be employed effectively to obtain the maximum mangiferin content for a variety of industrial applications.

Moreover, in the current investigation, mangiferin was recovered from the crude extract of *M. indica* leaves through purification. This phase attempted to eliminate contaminants (such as colorants and weakly polar molecules) that hindered the effectiveness of the extraction process and mangiferin’s biological activities [27]. Previously, employing macroporous HPD100 resin chromatography along with high-speed countercurrent chromatography (HSCCC), mangiferin was successfully isolated from Chinese mango cultivars [27]. To our knowledge, there have not been any established investigations on the purification of mangiferin from the leaves of *M. indica* growing in Eastern Province, Saudi Arabia. As a result, in this experiment, dichloromethane and ethyl acetate (liquid–liquid fractionation) and column chromatography were investigated as a promising method to separate mangiferin.

### 2.1. Structural Characterization of Mangiferin

**Figure 1:**
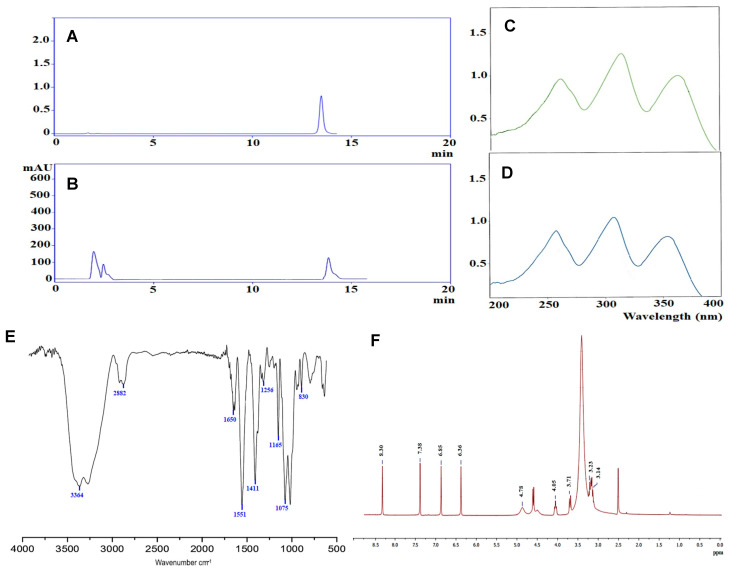
Structural elucidation of mangiferin isolated from *M. indica*. (**A**) HPLC pattern of standard mangiferin, (**B**) purified mangiferin. (**C**) UV–vis spectrum of standard, (**D**) mangiferin. (**E**) Mangiferin’s FTIR spectral analysis (4000–500 cm^−1^). (**F**) ^1^H NMR chemical shift (ppm) values in DMSO (400 MHz).

Figure 1 displays the findings of the HPLC analysis of purified mangiferin. It depicts three peaks with various RT values (Figure 1A). One of these peaks was shown at 13.97 min, which was comparatively close to the standard mangiferin peak that was observed at 14.12 min (Figure 1B). However, the peaks at 2.22 and 2.71 min were likely caused by mangiferin isomers such as homomangiferin and isomangiferin, which are slightly detectable in *M. indica* leaf extract [28]. Similar findings were conducted by Fernández et al. [29], which supported the identification of mangiferin in the crude mango leaf extract. Therefore, the level of the purified mangiferin was determined depending on the peak that appeared at 14.12 min.

The spectra of isolated mangiferin and the reference standard are shown using UV–visible spectrophotometry; mangiferin presented three significant peaks that were, respectively, 262, 314, 365, and 263, 315, 364 (Figure 1C,D). Notably, it had the same UV spectrum as the mangiferin reference standard. By superimposing the UV absorption spectra of the sample’s mangiferin bands with those of the reference, their identities were verified. Furthermore, by layering the UV absorption spectra of the resolved molecule obtained from the sample track at the start, middle, and end locations of the bands, the purities of the mangiferin bands were established. Interestingly, there was no interference from any other compound at the site where mangiferin was resolved because the bands’ identities and purities matched.

Mangiferin’s FTIR spectral data fell within the range of wavelengths 4000–500 cm^−1^, and the interpretation of the data was as follows: the peak at 3364 corresponded to alcohols and phenols (O-H stretch), the peak at 2882 accounted for alkane (C-H stretch), the peak at 1650 was related to the C=O stretch, the peak at 1551 corresponded to the aromatic C=C ring stretch, the peak at 1411 was associated with the –CH2 stretch, the peak at 1256 was assigned to the C-O-C stretch, the peak at 1165 accounted for the C-O stretch, the peak at 1075 corresponded to the RCH2OH O-H stretch, and the peak at 830 assigned to tetra-substituted aromatic bending (Figure 1E). The identity of the isolated mangiferin was validated by comparing the FTIR data to those of the reference mangiferin.

Data from the purified mangiferin’s ^1^H NMR (ppm), δ 8.30 (s, 1H, OH-1), δ 7.38 (s, 1H, OH-3), δ 6.85 (s, 2H, OH-6), and δ 6.36 (s, 2H, OH-7), were attributed to the aromatic proton of tetrahydroxy groups. The signals observed at δ 4.78, δ 4.05, δ 3.71, δ 3.23, and δ 3.14 strongly revealed the existence of a glucose moiety. The ^1^H NMR spectra of (Figure 1F) revealed distinctive peaks that corresponded to those previously described for mangiferin [30]. The sugar’s C-linkage was clearly visible because it did not exhibit the typical fragmentation signal for O-glycoside analogs. Such results, along with the chemical shifts of H-1 (δ 4.87), further supported this conclusion. The aglycon’s 1H chemical shifts were comparable with those for tetrahydroxyxanthones that have been reported in the literature [31]. The most extensively researched xanthone C-glucoside for pharmaceutical use is called mangiferin [32]. Likewise, it can be derived from a diverse range of plants, such as *Anemarrhena senkakuinsulare* (Aristolochiaceae), *A. asphodeloides* Bge, *Mahkota dewa*, and *Coffea pseudozanguebariae*. As a result, mangiferin contains hydroxyl substitutions at positions C-1, C-3, C-6, and C-7, as well as a glucose substitution at position C-2 of the xanthone skeleton. Taking into account mangiferin’s C-glycosidic bond that enhances bioavailability and is the cause of its antioxidant activities [33], it mimics the nucleophilic substitution of phloroglucinol. Moreover, it has been demonstrated that mangiferin has anti-inflammatory [34], antioxidant [35,36], antidiabetic [37], and anticancer [38,39] properties. 

We may conclude that mangiferin was successfully extracted based on the findings of UV, IR, NMR, and HPLC, so we moved on to evaluate its potential biological activities.

### 2.2. Antioxidant Activity

In order to inhibit or reduce the production of radicals and create less aggressive chemical species, which are more likely to trigger tissue injury, antioxidant activity includes intercepting reactive oxygen species [40]. Due to their capacity to defend against the negative effects brought on by reactive oxygen species, natural antioxidants have received a lot of interest in recent years [41,42]. In the DPPH assay, a purple solution that is visible receives electrons, changing it into a discolored solution [43]. The degree of change in color is related to the quantity and potency of the antioxidants present and shows the action of free radical scavengers [44].

**Figure 2:**
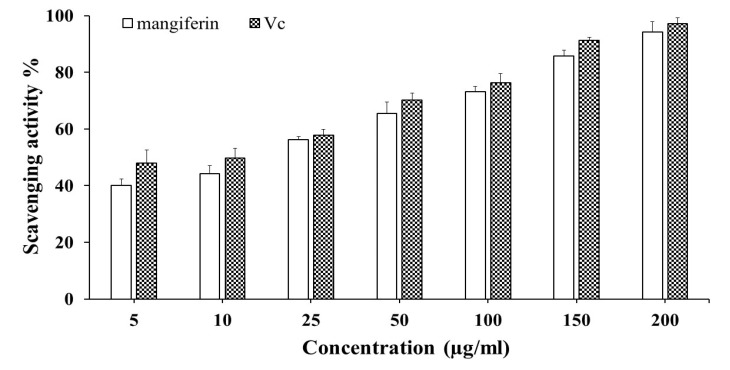
DPPH radical scavenging activity at different concentrations (5–200 µg/mL) of mangiferin and a positive control, vitamin C. Data are expressed as means ± SD, n = 3, and are displayed as a percentage of the control sample.

Antioxidants cause a drop in absorbance at 517 nm, which has been utilized to assess the DPPH free-radical scavenging activity [45]. The antiradical efficacy of isolated mangiferin in the current study demonstrated that treatments had a substantial impact. Figure 2 depicts the concentration-dependent scavenging pattern, indicating that with an increasing concentration, the DPPH radical scavenging activity increased, with the highest value of 94.2% recorded at 200 µg/mL, suggesting that the purified mangiferin was an efficient natural scavenger of free radicals. The higher DPPH values of isolated mangiferin were consistent with earlier studies by Crozier et al. [46], who found that the tested compounds’ values exhibited a linear relationship with polyphenols and recorded a DPPH activity ranging from 55 to 68.03% at concentrations between 10 and 50 mol/L. Moreover, polyaromatic polyphenolic compounds such as mangiferin have a stronger antioxidant activity, which is regulated by resonance energy, phenoxy radical delocalization, O-H bond dissociation, and steric hindrance, according to Rao and Gianfreda [47] and Rupasinghe et al. [48]. The purity and concentrations of mangiferin may affect its antioxidant capacity in this context [49].

In addition, the purified mangiferin displayed a convincing IC_50_ value of 17.6 µg/mL whereas Vc obtained a value of 11.9 µg/mL. When compared to other investigations, the mangiferin recovered from the current study had 2.18 times more free-radical scavenging activity than the mangiferin purified by a macroporous D101 resin (IC_50_ = 38.5 µg/mL) and by solvents with different levels of polarization (IC_50_ = 22.2 µg/mL) [50]. The existence of hydroxyl groups, which serve as metal chelators, and hydrogen donors neutralizers is undoubtedly significant [51]. Herein, many structural requirements for antioxidants were postulated based on the structure–activity correlations; the -OH in four positions on the ring had a remarkable antioxidant activity. The major structural feature for the efficacy of free-radical scavenging and the protective role on cells during oxidation is the presence of free OH groups. In addition, because of its aromatic nature and unique spatial structure, 2-phenyl substitution was found to be efficient for free-radical scavenging. It is commonly acknowledged that assessing plant-based extracts’ biological effects, such as their antioxidant properties, could give crucial information about how effective the processes of extraction and purification are. Consequently, based on the findings of the current investigation, it can be said that mangiferin with a high antioxidant potential was successfully generated using extraction, fractionation, and column chromatography.

### 2.3. Antimicrobial Activity

Due to drug abuse or overuse, pathogenic microbes in humans and animals are becoming more resistant to medications. There is a need for new, high-spectrum antibacterial medicines because they are particularly synthetic and can have negative side effects on users’ bodies [52]. In Table 1, the evaluation of the tested mangiferin’s antibacterial activity is shown using serial dilutions with a maximum concentration of 1000 µg/mL. Against *S. aureus*, it displayed considerable antibacterial action (MIC = 1.95 µg/mL), next against *E. coli* and *P. aeruginosa* (MIC = 7.81 µg/mL), and finally against *S. flexneri* (MIC = 62.5 µg/mL). The multidrug-resistant strains of *S. aureus* pose a significant challenge in hospital-acquired infections and are linked to a variety of illnesses, including deadly pneumonia, infective endocarditis, osteomyelitis, and minor skin and soft tissue infections [53]. According to studies, *M. indica*’s leaves and fruits may be utilized to manage skin conditions, including carbuncles, which are typically brought on by *S. aureus* infections. This is consistent with the findings of the current study [54,55]. According to research conducted by Teka et al. [56], the antistaphylococcal capabilities of *M. indica* bark and leaves, which contain the highest concentration of mangiferin, synergistically increased *S. aureus*’s vulnerability. These results add to a study by Mazlan et al. [57], who noted an improvement in antibacterial activity when mangiferin was combined with the antibiotics ciprofloxacin, nalidixic acid, and vancomycin to treat *S. aureus*. 

Bacteria can incorporate conjugates of siderophores and antimicrobial drugs into their cytoplasm or periplasm and need Fe^3+^ to proliferate [58]. According to Zhang et al. [59], the mangiferin molecule has a lengthy, nonpolar, hydrophobic alkyl saturated chain that may be helpful for membrane entry. Because of this property, the mangiferin compound would be a great siderophore-conjugating agent that could get past the outer membrane bilayer that protects bacteria. Future research on the synergistic interactions of mangiferin from *M. indica* with antibiotics may also result in the creation of novel antibacterial compounds.

The prevalence of fungi infections has increased to be one of the leading reasons for mortality and morbidity in patients with serious underlying illnesses, particularly those receiving treatment for hematological malignancies or staying in intensive care units [60]. The predominant *Candida* species are pathogenic fungi that could induce a number of symptoms, particularly in immunocompromised patients, ranging from cutaneous disorders to potentially fatal disseminated candidiasis [61]. Azole, polyene, and echinocandin are currently the three primary classes of antifungal medications. Even though antifungal medications are readily available, treating fungal infections is challenging due to a number of drawbacks, including a high cost, off-target toxicity, and ineffectiveness against drug-resistant strains in individuals receiving treatment [62]. As a result, the research and development of new antifungal therapies against *Candida* species are critical. Mangiferin, on the other hand, presented considerable anticandidal impact against all *Candida* species, with MIC values between 1.95 to 31.25 μg/mL, and the most sensitive to mangiferin were *C. albicans* and *C. glabrata*, with MIC values of 1.95 μg/mL, while *C. parapsilosis* and *C. tropicalis* were less so (MIC = 7.81 and 31.25 μg/mL, respectively) (Table 1), demonstrating a broad range of action that included the most common species involved with candidiasis. Thus, the findings of this investigation led to mangiferin as a prospective class for the development of antifungal therapeutic models. Undoubtedly, the introduction of a cationic group of mangiferin can significantly increase the potency against fungi and reduce the hemolytic activities. In addition, the antifungal properties of mangiferin, a polyphenol, could be attributed to its chemical structure and polarity, as the presence of polar groups reduces solubility and promotes diffusion across cell membranes. In this context, the existence of highly reactive hydroxyl groups as well as parts with a high affinity for attaching to proteins that predominantly block lipoxygenase and telomerase, as well as the interactions with the signaling transduction pathways of membrane receptors contribute to the activity of phenol compounds [63]. Cowan [64] highlighted, however, that in addition to these nonspecific interactions, a phenolic antifungal effect might also be achieved by inhibiting oxidative phosphorylation through the use of sulfhydryl groups.

### 2.4. Analysis of Time–Kill Profile

**Figure 3:**
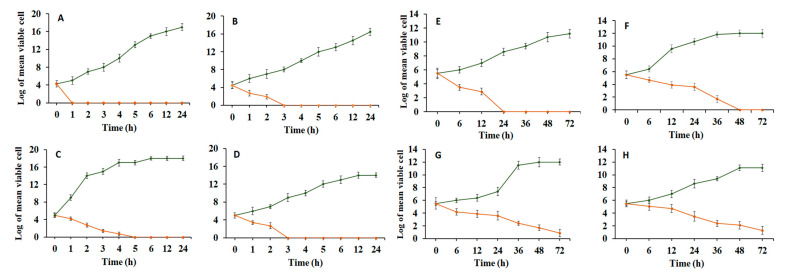
Mangiferin’s time–kill curve against different bacterial and fungal pathogens (**A**) *S. aureus*, (**B**) *E. coli*, (**C**) *S. flexneri*, (**D**) *P. aeruginosa*, (**E**) *C. albicans*, (**F**) *C. glabrata*, (**G**) *C. parapsilosis* (**H**) *C. tropicalis*, and untreated pathogen cells as growth controls. An overnight culture of selected strains were treated with the respective MICs of mangiferin and then incubated at 37 °C. Samples were gathered at the scheduled intervals and plated. Before counting the colony-forming units (CFU), the plates were incubated for 48 h.

Time–kill assays offer descriptive (qualitative) insights on the pharmacodynamics of antimicrobial drugs since the measurements are taken over different times; they have been commonly employed for in vitro investigations of new antimicrobial drugs. The time–kill kinetic patterns of mangiferin in the current investigation (Figure 3A) showed a fast bactericidal activity toward all susceptible strains. These results may be regarded as the first steps of the in vitro pharmacodynamics of *S. aureus*’s antibacterial activity because *S. aureus* was quickly killed by the MIC of mangiferin with a three-log decline in CFU/mL within 1 h. *S. flexneri* required 5 h to totally kill, whereas *E. coli* and *P. aeruginosa* were entirely killed in 3 h. These findings demonstrated that the antistaphylococcus action of the test compound’s MIC might effectively inhibit bacterial growth. To our delight, *C. albicans* was totally eradicated after 24 h of incubation, demonstrating the maximum fungicidal effectiveness of mangiferin. Further, it reduced the fungal load of *C. parapsilosis* and *C. tropicalis* by three logs after 72 h of incubation, whereas *C. glabrata* required 48 h. On the other hand, the growth control continued to develop throughout the trial, and no change in fungal burden was noted, validating our experimental conditions. Due to variations in the fungal cell wall, this phenomenon revealed that mangiferin had diverse antifungal effects against strains belonging to the same genus. The considerable reduction in cell viability at the MIC may be attributed to the fact that mangiferin is thought to be oriented toward DNA gyrase, therefore reducing DNA synthesis [65]. Furthermore, this finding was supported by an antimicrobial analysis and may have been impacted by the relationship between the structure of mangiferin and its activity. It was therefore hypothesized that the hydroxyl groups were essential to the antimicrobial effects of mangiferin because it allowed the compound to bind to bacterial lipid alkyl chains and enter the bacteria. According to strains, the cytoplasmic membrane integrity was disturbed by the mangiferin’s quick penetration into the bacterium, which led to the quick loss of intracellular components over a short period of time. The aforementioned findings taken together showed that mangiferin had a wide antimicrobial action and acted quickly in vitro to kill bacteria and fungi.

### 2.5. Cytotoxic Activity

Natural remedies have been demonstrated to be an effective and diversified sources of therapy for a range of human illnesses, particularly cancer [44]. Several *M. indica* cultivars’ whole fruit or fruit peel extracts had a toxic impact on cancer cell lines, including those from leukemia (Molt-4), colon (SW-480 and SW-620), lung (A-549), breast (MCF 7), and cervix (HeLa cells) cell lines. They were not harmful to the lung fibroblast’s normal cell line (CCD-25 Lu) and exhibited moderate cytotoxicity toward normal cell lines, such as colon (CCD-18Co) and breast (MCF-10A) [66,67,68] cell lines. The ability of natural extracts and purified compounds to inhibit cell proliferation and combat cancer can be quantified using the reliable and simple MTT method [42]. 

**Figure 4:**
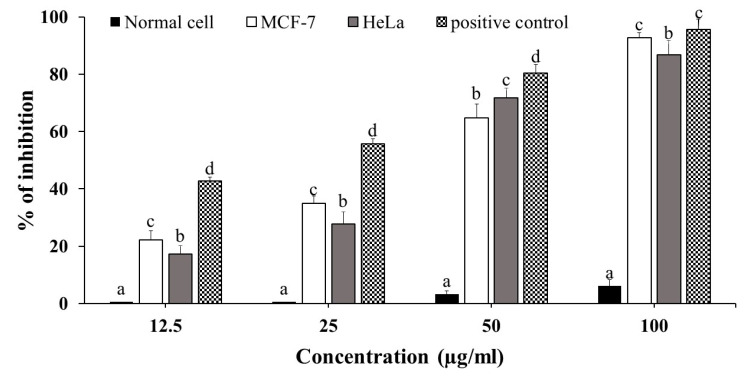
Cytotoxicity dose–response of mangiferin on the viability of MCF-7 and HeLa cell lines and Vero normal cell lines at various doses ranging from 12.5 to 100 µg/mL using the MTT test. The IC_50_ values were recorded to be 41.2 and 44.7 µg/mL for MCF-7 and HeLa, respectively. Different letters denote significant differences (*p* < 0.05) within the same concentrations of mangiferin. The results are represented by the mean ± SD of three independent experiments.

In this investigation, the mangiferin’s cytotoxicity on the MCF-7, HeLa, and normal NCM460 cell lines were presented as inhibition rate values. To our delight, even a very low dose of mangiferin (12.5 µg/mL) had an inhibitory impact on cell proliferation when added to the culture media of cell lines in a range of concentrations (12.5–100 µg/mL). The results indicated that all cancer cell lines were sensitive to mangiferin’s concentration-dependent cytotoxic action (Figure 4) as well as a positive control, doxorubicin. After 48 h of treatment, the vitality of MCF-7 cells tended to decline, with a mangiferin concentration of 22.2–92.7% and an IC_50_ of 41.2 µg/mL. Interestingly, mangiferin treatments had no effect on nontumorigenic cell lines; the only minimal viability effects were noted at high doses. However, when the HeLa cell line was exposed to various dosages of mangiferin, the antiproliferative inhibitory rate increased to 86.7% at 100 µg/mL, and the IC_50_ value was found to be 44.7 µg/mL. These findings implied that mangiferin had a rather high inhibitory effectiveness. Likewise, a number of cell lines, such as MCF-7 (IC_50_ = 18.9 µg/mL), KB (IC_50_ = 25.6 µg/mL), K-562 (IC_50_ = 25.4 µg/mL), K-562/Adr (IC_50_ = 24.7 µg/mL), and COLO205 (IC_50_ = 26.5 µg/mL), are affected by mangiferin’s ability to block cell proliferation [69,70]. 

The pharmacodynamic endpoint of anticancer drug therapy is apoptosis because this incidence confirms that cancer will not develop resistance to chemotherapy [71]. Additionally, apoptosis is an autonomous dismantling mechanism to eliminate specific cell components, and it minimizes damage to the healthy surrounding cells when cells undergo apoptosis, because it avoids the inflammatory response commonly associated with necrosis [72]. In order to further study whether the effective antiproliferative activity of the target mangiferin was related to the enhancement of cancer cell apoptosis in vitro, we performed a flow cytometry analysis on the MCF-7 cancer cell line and estimated the percentage of apoptotic cells using Ann/PI double staining. The target mangiferin was incubated with MCF-7 cells at various doses (100 and 200 µg/mL) for 48 h.

**Figure 5:**
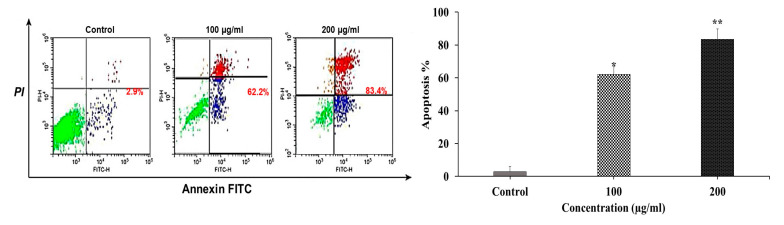
Induction of apoptosis by mangiferin in MCF-7 cell line, as shown by the annexin V-FITC and PI apoptosis analysis. Cells were treated with 100 and 200 μg/mL of mangiferin for 48 h. The results are presented as mean ± SD (n = 3). The data were analyzed utilizing a one-way ANOVA, followed by Dunnett’s post hoc test. Statistics were considered significant at *p* < 0.05. * *p* < 0.05 and ** *p* < 0.01 compared with the control.

In Figure 5, the lower left quadrant (Q4) of the figure shows living cells, the upper left quadrant (Q1) necrotic cells, the lower right quadrant (Q3) early apoptotic cells, and the upper right quadrant (Q2) late apoptotic cells. While PI denotes necrosis, annexin represents apoptosis. In order to be recognized by phagocytes during the early stages of apoptosis, the phospholipid phosphatidylserine (PS) is translocated from the inner to the outer layer of the plasma membrane. For the purpose of identifying early apoptosis, annexin V-FITC detects the evacuation of PS to the outer layer. Live cells and early apoptotic cells cannot pass through the nucleic acid-binding red fluorescent dye known as PI.

When the integrity of the cytoplasmic membrane is compromised, it can enter the nucleus and stain the DNA of cells that have undergone late apoptosis and necrosis. Interestingly, our data showed that mangiferin significantly and dose-dependently promoted early and late apoptosis in the MCF-7 cell line. The recognizable dot plot of Figure 5 shows the flow cytometric evaluation of apoptosis in comparison to the control, and the percentage of apoptosis in the MCF-7 cells treated with mangiferin significantly increased. Both 100 and 200 µg/mL values of apoptosis increased by 62.2 and 83.4%, respectively. Notably, the MTT assay results and the morphological observations caused by apoptosis were connected. Overall, the outcomes demonstrated that the mangiferin therapy prevented the MCF-7 cell line from proliferating by encouraging cell apoptosis. Many studies have demonstrated that bioactive substances, such as phenolic acid and mangiferin, contribute to the initiation and spread of cancer by controlling a variety of cellular functions, including DNA repair and the triggering of apoptosis [73]. Conversely, past studies on a range of cell types suggest that mangiferin suppress cancer cells’ proliferation by inducing cell death. In addition, mangiferin, for example, has been demonstrated to inhibit mitosis in human leukemia K562 cells, to restrict cell proliferation, and to initiate cell death [74]. Similar to this, it could block the proliferation of BEL-7404 human hepatocellular carcinoma cells by inducing apoptosis [75]. Thus, our observation of cell growth inhibition and induction of MCF-7 cell apoptosis is consistent with prior findings, suggesting that apoptosis induction may be one of mangiferin’s key anticancer mechanisms.

## 3. Materials and Methods

### 3.1. Plant Materials

From a nearby orchard in Eastern Province (Saudi Arabia), about 300 g of newly grown, dark-green, healthy leaves of *M. indica* (mango, local grafted type) were harvested in May 2022. A botanist from King Faisal University’s Biological Sciences Department authenticated the specimen, and a voucher specimen (BO 17706) was submitted in the herbarium. The leaves are spirally arranged on branches, linear-oblong, lanceolate-elliptic, and pointy at both ends, with leaf blades that are typically approximately 25 cm long and 8 cm wide but can occasionally be much larger. When the leaves are first formed, they are reddish and thinly flaccid, and when they are crushed, they release an aromatic odor. To avoid material deterioration, the collected leaves were rinsed three times with tap water, dried at 45 °C for 24 h, crushed into a fine powder measuring 1–2 mm in size, and then stored in zip-top bags at room temperature in a dry environment.

### 3.2. Extraction of Mangifera 

According to a description by Zou et al. [26], an ultrasonic bath (Bandelin RK 103H, Berlin, Germany) was implemented for the ultrasonic extraction method. The *M. indica* leaf powder (30 g) was accurately weighed and then dissolved in 60% ethanol in a capped glass vessel before being submerged in water in the ultrasonic device. Sonication was then conducted in accordance with the predetermined circumstances: liquid-to-solid ratios of 10/1 *v*/*w*, 60% ethanol concentration, 60 °C extraction temperature, and 4 min of extraction time. A 200 W electric power and 40 kHz frequency were set for this bath. After the extraction was performed, the sample was centrifuged for 15 min at 15,000 rpm to collect the supernatant. Under the same conditions, the precipitation was taken back and extracted again. 

### 3.3. Fractionation and Purification of Crude Extract

According to Singh et al. [76], the crude leaf extract was treated by liquid–liquid fractionation along with column chromatography for the fractionation and purification. In a nutshell, it was diluted in 60% ethanol and extracted three times for 24 h with dichloromethane (1:1 *v*/*v*), resulting in upper and lower layers. The bottom layer and top layer were then created by continually extracting the upper layer three times for 24 h with ethyl acetate (1:1 *v*/*v*). The absorbance of the layers was then monitored at a wavelength of 318 nm. Both layers were collected; using a glass column loaded with silica gel 60 (0.04–0.06 mm) as the stationary phase and chloroform/ethanol with changing polarity (90:10 to 50:50 *v*/*v*) as the mobile phase, the analysis was performed for the layer which had a high mangiferin content. The contaminants were then removed using methanol and acetone [77] before the mangiferin crystals were obtained (468 mg) from the vacuum evaporation. The purified mangiferin underwent an HPLC analysis.

### 3.4. High-Performance Liquid Chromatography Analysis

The HPLC spectrum of the purified mangiferin was determined, following the protocol in [78] with some adjustments. In brief, 1 mg of standard mangiferin (Sigma-Aldrich, Steinheim, Germany) and 0.095 g of ethyl acetate fraction were precisely weighed. Following that, each sample was diluted in 60% ethanol and run through a 0.22 µm nylon filter (Merck KGaA, Darmstadt, Germany). An Agilent 1260 Infinity II HPLC with a UV–vis detector and a C-18 column (25 cm × 4.6 mm × 100 mm) (Kunaer, Germany) were used to identify the presence of purified compound. In the mobile phase, acetonitrile and acetic acid at 0.5% (1:1 *v*/*v*) were utilized. The temperature was 30 °C, and the flow rate was 0.8 mL/min. In a total volume of 20 µL, each prepared sample was injected onto the column. The detection wavelength was 318 nm. Claritychrom software (V7.4.2.107, Santa Clara, CA, USA) was used to analyze the chromatograms. The content and retention time (RT) of mangiferin were estimated using a standard curve created from reference mangiferin.

### 3.5. Structural Clarification of Mangiferin

UV–vis, FTIR, and ^1^H NMR spectra were gathered to explore the physicochemical features of mangiferin.

#### 3.5.1. UV–Visible Spectroscopy 

The spectral scanning of the isolated mangiferin at wavelengths between 200 and 400 nm was carried out by a UV–visible spectrophotometer (UV-1800–240V Shimadzu, Koyoto, Japan). The isolated mangiferin was prepared by dissolving it in methanol at a dose of 40 µg/mL. Reports were made comparing the spectra of the target compound and their reference standard.

#### 3.5.2. Fourier Transform Infrared Spectroscopy 

Using the potassium bromide (KBr) pellet technique in an FTIR spectrometer (Nicolet 5700, Thermo Fisher Scientific, Madison, WI, USA), one milligram of the isolated mangiferin crystals was analyzed. The prospective compound’s IR data were compared to the mangiferin reference standard in the range of 4000–400 cm^−1^.

#### 3.5.3. Nuclear Magnetic Resonance Spectroscopy

On a Bruker DRX 500 NMR equipment (Bruker, Ettlingen, Germany) operating at 400 MHz at 25 °C, ^1^H NMR spectra were captured. For ^1^H, a range from 0 to 9 ppm was used. Tetramethylsilane (TMS, Merck, Darmstadt, Germany), the internal standard, was used to calibrate the signals. To record the spectra, 10 mg of the sample was dissolved in 0.5 mL of DMSO.

### 3.6. Biological Activities

#### 3.6.1. 1,1-Diphenyl-2-picrylhydrazyl Radical Scavenging Assay

Purified mangiferin’s antioxidant activity was assessed using Almustafa and Yehia’s [42] technique with a few minor modifications. Briefly, a fresh solution of 0.1 mM DPPH (1,1-Diphenyl-2-Picrylhydrazyl) was obtained by dissolving 1.9 mg in 1000 mL of 99.7% ethanol. To 2.4 mL DPPH solution, different concentrations of the sample (1.6 mL, 5, 10, 25, 50, 100, 150, and 200 µg/mL) were mixed. After giving the reaction mixtures a gentle shake, they were let stand at room temperature for 30 min. Using a UV–vis spectrophotometer (Shimadzu, Tokyo, Japan), the samples’ absorbance at 517 nm was evaluated. The negative control was pure 60% ethanol, whereas vitamin C (Vc) served as the positive control. The amount of an antioxidant that caused a 50% inhibition of the oxidant IC_50_ value was calculated. The following formula was used to estimate the DPPH scavenging activity (in %):

Scavenging activity % = Aa (control) − Ab (sample)/A (control)

where Aa is the absorbance of the DPPH mixture and the blank; Ab denotes the absorbance of the sample and the DPPH.

#### 3.6.2. Antimicrobial Assay

##### Inoculum Preparation

*Candida tropicalis*, *C. parapsilosis*, *C. glabrata*, and *C. albicans* were cultured in accordance with the Clinical Laboratory and Standard Institute document M27-A3 (CLSI 2008) [79]. Sabouraud dextrose (SD, Acumedia, San Bernardino, CA, USA) agar was utilized to cultivate *Candida* strains for 48 h, and isolated colonies were then suspended in 0.85% NaCl saline solution (Synth, Diadema, SP, Brazil). The resulting suspension’s density was adjusted to the 0.5 McFarland standard or 10^6^ colony-forming units (CFU)/mL. After that, an SD broth (Acumedia, USA) was used to dilute the fungal solution to a concentration of 1 × 10^3^ CFU/mL. However, *Shigella flexneri*, *Pseudomonas aeruginosa*, *Escherichia coli*, and *Staphylococcus aureus* inoculum suspensions were prepared in nutrient broth (NB, HiMedia, Mumbai, India) for 24 h at 37 °C. Following incubation, a sterile Ringer solution was used to dilute each strain to a final concentration of 10^5^ cells/mL. All test strains were kindly obtained from King Abdulaziz University, Department of Microbiology, Saudi Arabia.

##### Determination of Minimum Inhibitory Concentration 

MICs were established using the broth microdilution technique [80,81]. Mangiferin’s antimicrobial activity against the pathogenic bacterial and fungal strains was assessed via MIC values. Briefly, on a 96-well plate, the purified compound was dissolved in DMSO (1 mg/mL, Sigma-Aldrich, St. Louis, MO, USA) and diluted at concentrations of 1000–0.49 μg/mL by two-fold dilutions. Each pathogen suspension (100 μL) was injected into each well with 100 μL of the tested compound, and the mixture was then incubated for 48 h at 37 °C. The lowest concentrations of the compound that prevented microbial growth were recorded as the MIC values at the end of the incubation time. Parallel tests were conducted using DMSO as the solvent control; in addition, amphotericin B^®^ and ciprofloxacin (Sigma Chemical Co., St. Louis, MO, USA) were included as the positive controls. All tests were conducted in triplicate. 

#### 3.6.3. Time–Kill Assay

A time–kill assay was used to measure how well the tested compound killed bacteria and fungi. The investigation was conducted using the viable cell count method that Kaur and Arora [82] described, with a few changes. Tubes with 10 mL of broth media containing an inoculum suspension at a concentration of 1 × 10^6^ CFU/mL and the tested compound at doses equivalent to the MIC were incubated at 37 °C. Then, approximately 100 μL of samples were collected from each treatment at 0, 1, 2, 3, 4, 5, 6, 12, and 24 h for bacteria. In contrast, samples of fungi were taken at 0, 6, 12, 24, 36, 48, and 72 h, plating directly onto RPMI 1640. After a 48 h incubation period, the viable colonies were monitored. DMSO was utilized as a solvent control. After each trial was performed in triplicate, the findings were scrutinized and graphically represented. A graph of the log CFU/mL against time was constructed.

#### 3.6.4. Cytotoxic Assay

The cervical carcinoma (HeLa) and breast carcinoma (MCF-7) cell lines, as well as the healthy human intestinal epithelial cell line (NCM460 cell), were provided by Shanghai Bioleaf Technology Co., Ltd., Shanghai, China. As previously described by [42] with minor changes, the cell viability was ascertained using a 3-(4, 5-dimethylthiazol-2-yl)-2,5-diphenyltetrazolium bromide test (MTT; Sigma-Aldrich, USA). The cells were maintained in Dulbecco’s modified Eagle’s medium (DMEM, Life Technologies, Gaithersburg, MD, USA) containing 10% fetal bovine serum (FBS, Gibco, Massachusetts, MA, USA), 50 µg/mL streptomycin, and 1% penicillin (Sigma-Aldrich, St. Louis, MO, USA). All cell lines were incubated at 37 °C, an environment that was humidified and contained 5% CO_2_ (NuAire incubator, Thermo, Forma 370). A 96-well culture plate was seeded with cells at a density of 1 × 10^5^ cells/well and then incubated for 2 h to synchronize until 80% of the cells had fused. The target compound (10 µL, 12.5, 25, 50, and 100 µg/mL) or doxorubicin—a positive control—was incubated with the cancer cells for 48 h. The test’s negative control consisted of wells with cells without treatment. After 4 h at 37 °C of incubation, we added 20 µL (5 mg/mL) of MTT reagent to each well. Then, after the medium was removed, we thoroughly agitated the plate for 1 h. To dissolve the formazan crystals, 150 µL of DMSO (0.1% *v*/*v*) and 25 µL of glycine (0.1 mol/L) were applied to each well. We assessed the absorbance at 570 nm by employing a microplate reader (Infinite 200 Pro, Tecan, Männedorf, Switzerland). The compound’s cytotoxicity was measured as a percentage of inhibited cell growth utilizing the following formula: cell viability (%) = (drug OD 570 nm/control OD 570 nm) 100%. A graph showing the percentage of cell mortality versus compound concentrations was created to calculate the concentration of the compound that caused 50% of cell death, or the inhibitory concentration (IC_50_). 

#### 3.6.5. Cell Apoptosis Analysis

In accordance with the manufacturer’s guidelines, cellular apoptosis was evaluated using the KeyGEN Biotech Apoptosis Assay Kit (Southern Biotech). In a nutshell, a 6-well plate containing 1 × 10^6^ MCF-7 cancer cells was incubated for 12 h before being treated with a compound at doses of 100 and 200 µg/mL. We harvested the cells after 48 h and then washed them three times in ice-cold PBS. Following that, 5 µL of annexin V-FITC (fluorescein isothiocyanate) was mixed with 100 µL of cell aliquots and left to incubate for 15 min in the dark. Then, 5 µL of PI (propidium iodide) buffer was added after staining, carefully mixed, and maintained on ice. After the samples were examined by a Beckman DxFlex flow cytometer, singlet cells were displayed as FITC-A against PI dot plots. The percentages of viable cells (annexin V low, PI low), early proapoptotic cells (annexin V high, PI low), and late apoptosis/necrotic cells (annexin V high, PI high) were detected using quad gates.

### 3.7. Statistical Analysis

All investigations were conducted in triplicate, and the results were represented by the mean ± standard deviation of three independent trials (n = 3). The data were subjected to a one-way analysis of variance (ANOVA) followed by Tukey’s multiple comparison test. *p*-Values < 0.05 were considered significant.

## 4. Conclusions

Pathogens are now managed by chemical and physical agents, which may be harmful to the environment as well as animal and human health. As a result, the separation and identification of bioactive components from natural sources is critical for the development of new therapeutics. Mangiferin, one of the primary components of *M. indica*, contributes to a number of positive biological processes in the plant. Using an efficient purification approach that included a liquid–liquid separation and column chromatography, 468 mg of pure mangiferin was obtained from *M. indica* leaves. The structure of mangiferin was determined using a variety of spectroscopic data. Surprisingly, the in vitro results demonstrated that mangiferin had a considerable antioxidant efficacy against DPPH free radicals, with an IC_50_ value of 17.6 µg/mL. It did, however, show a good antibacterial action against *P. aeruginosa*, *S. aureus*, *E. coli*, and *S. flexneri* with MIC values ranging from 1.95 to 62.5 µg/mL. It also showed a robust anticandidal action against *C. glabrata*, *C. albicans*, *C. parapsilosis*, and *C. tropicalis*, with MIC values ranging from 1.95 to 31.25 µg/mL. According to cytotoxic and flow cytometric tests, a potent mangiferin exhibited a good cytotoxic efficiency against HeLa and MCF-7 cell lines. Furthermore, mangiferin demonstrated a negligible cytotoxicity against normal cells, indicating that it was safe for normal cells and a strong candidate for use as an anticancer therapeutic. The outcomes of this research shed light on the possibilities of employing *M. indica* leaves as a source of mangiferin. Undoubtedly, it has the potential to be used as a medicinal agent, an antibacterial, and an antioxidant natural agent. Further investigation on the molecular mechanism of the antitumor action is required.

## Figures and Tables

**Table 1:** Minimum inhibitory concentration (MIC) values (µg/mL) of the mangiferin isolated from *M. indica* leaves against bacterial and fungal pathogenic strains. Ciprofloxacin and amphotericin B were the positive controls.

Bacterial strains (MICs)				
Ciprofloxacin	*E. coli*	*P. aeruginosa*	*S. aureus*	*S. flexneri*
0.49	7.81	7.81	1.95	62.5
Fungal strains (MICs)				
Amphotericin B	*C. albicans*	*C. glabrata*	*C. parapsilosis*	*C. tropicalis*
0.49	1.95	1.95	7.81	31.25

## Data Availability

The data that support the findings of this study are available from the corresponding author upon reasonable request.
